# Multivariate path analysis of the relationships between seedling regeneration and environmental factors beneath a dwarf bamboo understory

**DOI:** 10.1002/ece3.5548

**Published:** 2019-09-03

**Authors:** Feng Qian, Haiyan Song, Miao Chen, Jiaqin Zeng, Chenqiang Dang, Jianping Tao

**Affiliations:** ^1^ Key Laboratory of Eco‐environments in Three Gorges Reservoir Region (Ministry of Education) Chongqing Key Laboratory of Plant Ecology and Resources Research in Three Gorges Reservoir Region School of Life Sciences Southwest University Chongqing China

**Keywords:** bamboo density, dwarf bamboo, environmental factors, path analysis, seedling emergence, seedling establishment

## Abstract

Seedling emergence and establishment are fragile processes that determine the direction and structure of forest succession and regeneration. However, seedling emergence and establishment are easily affected by biotic and abiotic (environmental) factors. A dense and expanding understory of dwarf bamboo is one such important factor that can seriously hinder the seedling regeneration. We conducted a field experiment to investigate the emergence and establishment of canopy tree seedlings under artificially controlled densities of dwarf bamboo. We found that understory dwarf bamboo obstructed seedling emergence but reduced the death of seedlings. Although understory dwarf bamboo reduced the median retention time of seedlings, dense bamboo increased the mean survival time of seedlings. Our results suggest that understory dwarf bamboo has multiple selectivities for tree seedling emergence and establishment: high‐density dwarf bamboo was beneficial to evergreen species but lower‐density of bamboo was conducive to the survival of deciduous species, it means the dwarf bamboo potentially alters successional trajectories of forest communities. Path analysis revealed that the most important factors affecting tree seedling emergence and death were the abundance of seeds in the seed bank and the density of emerged seedlings, and that the soil temperature promoted seedling emergence but increased seedling death, the thickness of litter limited seedling emergence, and the leaf area index of the bamboo canopy limited seedling death. The present study suggests that dwarf bamboo can directly alter the microenvironment, significantly reducing light levels and soil temperature but increasing the thickness of litter and soil humus, thereby indirectly impacting the regeneration of tree seedlings. Our results indicate that various factors affected seedling emergence, and there were complex indirect relationships among these factors. In general, biological factors had a stronger influence on tree seedling regeneration than environmental factors.

## Graphical abstract

Understory dwarf bamboo is expanding rapidly and resulting in irregular bamboo shrub layer formation in forests. This was not only can alter the ground surface environment (such as light, thickness of litter, thickness of soil humus, soil temperature, and soil humidity), but also slowed down the speed and density of seedling emergence, and accelerated the rate of seedling death; it has inhibited the establishment of local woody species, acting as an “ecological filter” in forest regeneration. And the multivariate path analysis showed that seed bank and soil temperature were the most important decision factors affecting seedling emergence, and the density of emerged seedling was the decision factor for seedling mortality, exerting a density dependence effect, while the litter thickness, leaf area index with dwarf bamboo canopy and dwarf bamboo density were the key limitation factors for seedling mortality, and soil temperature is the important promoting factor for seedling death.

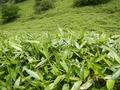

## 1 INTRODUCTION

Natural forest regeneration refers to the continuous process by which plant species generate seeds that germinate in soil and grow to become mature specimens or communities (Harper, 1977). The establishment of seedlings is a sensitive and critical part of the regeneration process that not only affects population dynamics and distribution patterns but also controls succession in forest communities (Aguilera & Lauenroth, 1993; Hirobe et al., 2017). However, seedling establishment can be easily perturbed by biotic factors (such as seed sources and animal predation) and by abiotic or environmental factors (such as light, moisture, and temperature). The understory vegetation, such as ferns and dwarf bamboos, also influences seedling regeneration (George & Bazzaz, 1999; Giordano, Sánchez, & Austin, 2009). Understory plants are generally shade‐tolerant and fast‐growing (Figueroa & Lusk, 2001) and readily form a thick shrub layer in forests (Wang, Tao, & Zhong, 2009). In recent years, disturbances and browsing regimes can result in large increases in the density and cover of understory clonal vegetation (Mallik, 2003). In many cases, these species expand to form persistent, monodominant layers that can be nearly impenetrable; once this layer has formed, it can remain intact for decades even in closed‐canopy forests (Royo & Carson, 2006).

Dwarf bamboo, a perennial evergreen, is broadly distributed in the understory of subalpine forests and some subtropical mountain forests in southwest China, Japan, and South America (Kudo, Amagai, Hoshino, & Kaneko, 2011; Lima, Rother, Muler, Lepsch, & Rodrigues, 2012; Taylor, Zisheng, & Jie, 1996; Wang et al., 2009). Because of its clonal growth characteristics and root‐whip system, dwarf bamboo is highly plastic and adapts rapidly to environmental changes. When exposed to relatively high levels of light and temperature, dwarf bamboo demonstrates high rates of growth and vegetative expansion. Therefore, with global warming, dwarf bamboo has expanded significantly in japan and southwest of China, colonizing forest gaps and understories and gradually become the dominant understory layer in many regions (Kudo et al., 2011; Lima et al., 2012; Montti, Villagra, Campanello, Gatti, & Goldstein, 2014; Royo & Carson, 2006; Wang, Shi, & Tao, 2012; Taylor et al., 2006; Wang et al., 2009).

Understory dwarf bamboo has a strong foraging ability on resources and it will be fierce competition with competes fiercely with other species for resources and resists displacement (Darabant, Rai, Tenzin, Dorji, & Gratzer, 2005). It is commonly regarded as an “ecological filter” for forest regeneration (Itô & Hino, 2007; Takahashi, Mitsuishi, Uemura, Suzuki, & Hara, 2003; Takahashi, Uemura, Suzuki, & Hara, 2003; Takanishi, Uemura, & Hara, 2002). Seedling regeneration is hindered by dwarf bamboo (Doležal, Matsuki, & Hara, 2009; Giordano et al., 2009; Iida, 2004; Montti et al., 2011) and is lower in plots with bamboo than in bamboo‐free plots (Itô & Hino, 2007; Narukawa & Yamamoto, 2002). The effects of dwarf bamboo on seedling regeneration are thought to be due to the way that the dense layer affects the ground surface microenvironment. The clumped stem growth, intertwined bamboo rhizomes, and dense foliage block most of the light (Kobayashi, Shomano, & Muraoka, 2004; Zuzana & Tomas, 2005), increase soil moisture, reduce soil temperature (Takahashi, Mitsuishi, et al., 2003; Takahashi, Uemura, et al., 2003; Takanishi et al., 2002), and delay litter decomposition, leading to a thicker litter barrier layer in bamboo‐covered stands (Doležal et al., 2009; Watanabe, Fukuzawa, & Shibata, 2013).

In the field, environmental factors are complex and intertwined, and a little is known about the effects of temperature (Edwin, Randall, & Vandegenachte, 1996; Kyereh, Swaine, & Thompson, 2010), light (Elliott & White, 1994; Zheng, Xie, Gao, Jiang, & Tobe, 2004; Zuzana & Tomas, 2005), water stress (Kyereh et al., 2010), litter layer (Doležal et al., 2009; Peterson & Facelli, 1992; Watanabe et al., 2013), bamboo density, and combinations of these factors on seedling regeneration. The seed source is an additional key factor for seedling regeneration, especially in natural forest communities, because seed production varies in dwarf bamboo stands of different densities (F. Qian, H. Y. Song, J. P. Tao, unpublished data). However, using path analyses to take these factors into account together and found out which factor(s) was the main influence on seedling regeneration was very important. Meanwhile, several important and intractable issues remain to be studied: which factors form the main filter for seedlings in the natural regeneration of forest communities, and how much influence do these factors have?

The purpose of the present study was to address these questions by simulating different bamboo densities (relative density: 0%, 25%, 50%, and 100%) by artificially controlling the number of bamboo culms in field plots, and then monitoring the regeneration dynamics (emergence and survival) of canopy tree seedlings in these plots. Our goals were as follows: (a) To determine how differences in the density of dwarf bamboo influence the dynamics of tree seedling emergence and survival; (b) To probe the relationships between tree seedling regeneration and the various changes to the microenvironment associated with the dwarf bamboo understory; (c) To use path analysis (with biotic and abiotic factors as potential independent variables, and seedling emergence and death as dependent variables), to identify the key factor(s) for seedling regeneration, and to quantify the effect of these factor(s). We hypothesized that an understory of dwarf bamboo selectively filters seedling emergence and survival, by altering the understory environment, thus determining seedling dynamics and abundance. This study is the first to assess the effects of combinations of factors on seedling regeneration, and the first to apply path analysis to identify the most critical factors. This study reveals the indirect influence of factors at the community level and provides an effective theoretical reference for future forest management.

## 2 METHODS

### 2.1 Study site

The study was carried out in the Jinfo Mountain National Nature Reserve (28°46′−29°38″N, 106°54′−107°27″E, altitude ~1,450 m), Chongqing, China. Jinfo Mountain runs from west Hubei to east Sichuan and is located at the boundary of four major plains (Yi, Huang, Xiao, & Quan, 2012), the East and West plains, and the North and South plains. The reserve covers an area of 1,300 km^2^ at altitudes ranging from 340 to 2,251 m. Mount Jinfo is predominantly composed of limestone and has typical Karst geomorphic landforms. The region has a subtropical humid monsoon climate: the annual average precipitation is 1,395.5 mm, the average amount of sunshine is 1,079.4 hr, and the relative humidity is 90%. We selected evergreen and deciduous broad‐leaved mixed forest areas for our study. The soil type in the study blocks was yellow brown soil (Zhang, 2014, Masters Dissertation). The forest canopy in the study areas is dominated by *Castanea henryi* (Skan) Rehd. et Wils., *Rhododendron faberi* Hemsl., *Symplocos setchuensis* Brand, *Elaeocarpus japonicus* Sieb. & Zucc., *Acer davidii* Franch., *Elaeocarpus sylvestris* (Lour.) Poir, and *Carpinus viminea* Lindl. The understory vegetation is dominated by *Fargesia decurvata* J. L. Lu (height ~ 0.80 m, crown size = 0.4 cm × 0.4 cm), *Chimonobabusa utilis* (Keng) Keng f. (height ~ 3.12 m), and *Qiongzhuea communis* Hsueh (height ~ 1.51 m). Because of climate change, dwarf bamboo coverage in the Jinfo Mountain reserve is expanding, commonly forming a dense stratum near the forest floor (Qian, Zhang, Guo, & Tao, 2016). For more detailed site information, please see Table 1.

**Table 1:** Stand characteristics of the three experimental sites in Jinfo Mountain National Nature Reserve, Chongqing, China

Stand characteristic	Site A	Site B	Site C
Altitude	1,450 m	1,458 m	1,453 m
Longitude and latitude	29°49′49.30″N 107°09′32.26″E	28°59′39.04″N 107°09′10.51″E	28°59′52.01″N 107°09′59.95″E
Forest type	Evergreen and deciduous broad‐leaved mixed forest		
Overstory vegetation	*Castanea henryi* (Skan) Rehd. et Wils., *Rhododendron faberi* Hemsl., *Symplocos setchuensis* Brand, *Elaeocarpus japonicus* Sieb. & Zucc., *Acer davidii* Franch., *Elaeocarpus sylvestris* (Lour.) Poir, and *Carpinus viminea* Lindl., etc.		
Understory vegetation	*Fargesia decurvata* J. L. Lu, *Symplocos henryi* Brand, *Carpinus viminea* Wall., etc.		
Soil type	Yellow brown soil	Yellow brown soil	Yellow brown soil
Slope	9°	8°	9°
Aspect	South‐southeast 23 degrees	South‐southeast 24 degrees	South‐southeast 23 degrees
Average annual temperature	14.5°C (air temperature)		
Average annual rainfall	1,395.5 mm		

### 2.2 Experimental manipulations

The criteria for study site selection were as follows: (a) sites should have a relatively flat topography; (b) blocks should represent the forest structure and species composition present in the study area; (c) block habitats should have little evidence of human disturbances, such as strip cutting; (d) the herb/shrub stratum in blocks should be categorically dominated by high‐density and uniformly distributed clonal dwarf bamboo *F. decurvata*; and (e) the species composition of the overstory should be very similar among the selected blocks.

**Figure 1:**
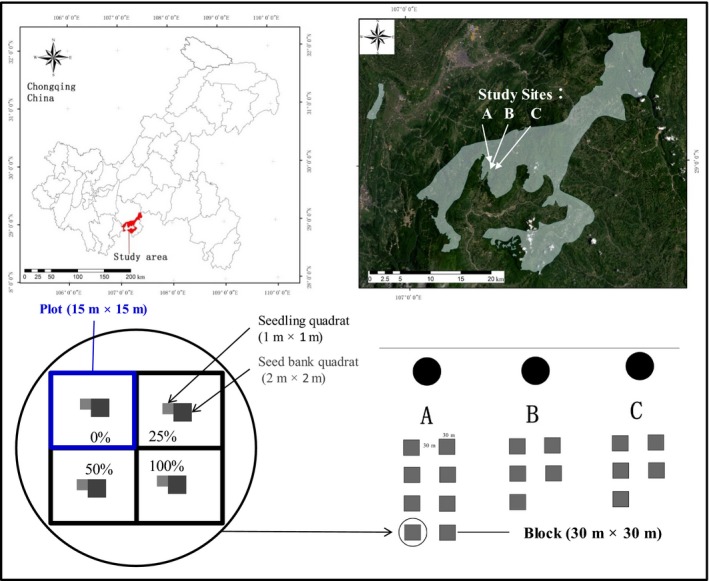
Geographical location of study sites in Chongqing, southwest China. The three selected sites (A, B, and C) totalled ~1.8 ha of forest. Site A was divided into eight blocks (each block = 30 m × 30 m), and sites B and C were divided into five blocks (30 m × 30 m).The spacing between blocks was not less than 30 m. Each block was divided into four plots (treatments; 15 m × 15 m). The relative density of dwarf bamboo was artificially maintained at 0%, 25%, 50%, and 100% in the four plots. A seedling quadrat (1 m × 1 m) and a seed bank quadrat (2 m × 2 m) were placed in the central of each plot

After reconnaissance of the study area in late August 2012, we selected three representative study sites (A, B, and C) on Mount Jinfo. We set up 8, 5, and 5 blocks in sites A, B, and C, respectively. Each block was 30 m × 30 m, and the distance between blocks was 30–45 m. Each block was divided into four treatment plots (15 × 15 m). Next, one seedling quadrat (1 m × 1 m) and one seed bank quadrat (2 m × 2 m) were positioned in the central area of each plot (treatment). Therefore, a total of 72 seedling quadrats and 72 seed bank quadrats were set up (Figure 1).

Treatments: we counted the number of dwarf bamboo culms in the densest area (1 m × 1 m, *n* = 30) in three study sites and used this value to represent 100% relative bamboo density in the study. Using the method of artificial symmetrical removal of dwarf bamboo, we created four treatments in each block with relative densities of 100% (76.29 ± 6.51 culms per m^2^), 50% (38.02 ± 3.51 culms per m^2^), 25% (19.01 ± 1.85 culms per m^2^), and 0% (no bamboo). Seedlings, herbs, and liana were also removed. To control the density of dwarf bamboo, we regularly removed newly established dwarf bamboo.

#### 2.2.1 Seed bank for seedling regeneration

Seeds are the material basis for seedling emergence, and the seed bank is the most direct and effective source of seeds. Therefore, we investigated the effective soil seed bank demography in plots with different densities of bamboo. Prior to the start of the study, the initial litter layers and seeds were removed from the seed bank quadrats, and soil samples were collected from each seed bank quadrat in March (before natural seed germination) and November (after new seed input) from 2013 to 2016. One soil sample (size: 0.5 m × 0.5 m) was randomly and carefully excavated from each quadrat from the top 10 cm of soil plus litter layer. Because we wanted to count only the seeds from canopy trees (which are larger than 1 mm × 1 mm) and exclude herbaceous seeds, we separated tree seeds from soil samples using the floating method. (a) Each soil sample was placed in a sieve with an aperture of 0.5 mm. (b) The sample was washed with water to remove soil, leaving seeds, stones, litter, and roots. (c) Samples were air‐dried and seeds were separated. (d) Litter samples were dried naturally and the seeds separated. (e) To identify seed activity, we directly examined the embryos and recorded the endosperm color of each seed to determine whether it was viable or rotten. If the endosperm was brown, rotten, or otherwise unhealthy, the seed was deemed inactive and unable to germinate; if the endosperm was white and full (healthy), the seed was considered active (Qian et al., 2016). We determined the species composition and abundance (individuals per m^2^) for active seeds in all plots of differing bamboo density.

#### 2.2.2 Dynamics of seedling emergence and survival

Seedling emergence and survival censuses were initiated on 15 September 2012 in each seedling quadrat, and repeated at ~15‐day intervals during the growing and drought seasons (March to August), and monthly during the other seasons. Each newly emerged seedling was identified at the species level and marked with a plastic tag to facilitate recording, with tags recording species, emergence date, death date, and the seedling number. Emergence was scored as the visibility of cotyledons or leaves above the litter layer. Dead seedlings with symptoms such as rotten or dry stems, roots entirely exposed by soil erosion, or death due to fallen branches or gravel were also recorded (Dai, Seiwa, & Sakai, 2002). Seedling survival time was the time between seeding appearance and death (e.g., seedling X, emerged on 1 October 2013, died on 1 October 2014; seedling survival time = 365 days). Herbaceous plants were not recorded. Unidentified seedlings were allowed to grow until the species could be identified. To compare species composition in the aboveground vegetation and in the seed bank, the coverage of each species of seed plant was estimated visually in each plot. This study was continued until 17 December 2016.

### 2.3 Environmental factors

Environmental conditions associated with the experimental manipulation were measured in all plots at the same frequency as the seedling survey. The leaf area index was evaluated with LAI‐2000 photosensitive light sensors (LI‐COR Inc), with one light sensor per measurement period placed in the same vertical location in each quadrat, 10 cm above the ground surface or dwarf bamboo crown. The thickness of the litter and humus was measured with a ruler to the nearest mm at 7–10 random points in each plot. Soil temperature and soil humidity were measured with a Hydra Probe II (HPII, Stevens W.M.S. Inc.).

### 2.4 Statistical analysis

In this paper, we aimed to elucidate the regeneration of seedlings at the community level, rather than at the species or individual level. Therefore, for our initial analysis, we converted the seed and seedling numbers to seed or seedling density (individuals per m^2^ per year). Differences in the overall density of seeds in bamboo plots of differing density were analyzed by a one‐way analysis of variance (ANOVA) followed by least significant difference (LSD) multiple comparison tests when the ANOVA was significant. The effects of bamboo density on overall seedling density, seedling emergence rate, seedling death rate, and environmental factors were assessed with the same analytical method. We also performed a survival analysis using the life tables method (Wilcoxon: Gehan) to test the effect of dwarf bamboo density on the median seedling retention time (speed of death) and the mean seedling survival time. The statistical analyses were conducted in SPSS 13.0 (SPSS).

#### 2.4.1 Path analysis

**Figure 2:**
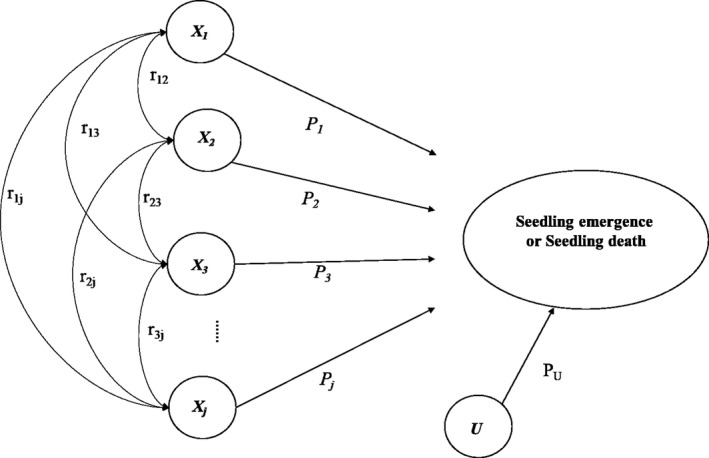
Path diagram illustrating the paths by which the independent variables *X*_1_…*X_j_* (environment factors, seed bank density, bamboo density) influence seedling emergence and death. The environment variables consisted of the leaf area index of the forest canopy (LAI), the leaf area index of the dwarf bamboo canopy (LAID), the thickness of litter (THL), the thickness of soil humus (THSH), the soil temperature (ST), the soil humidity (SH), and the residual (*U*). *P_j_* = direct path coefficients, with the thickness of the arrows representing the strength of the relationship. *r_ij_* = correlation coefficients between the independent variables

Path analysis is based on the decomposition of correlation coefficients into direct and indirect effects and the complete determination of a variable by other variables (Kozak & Kang, 2006). The direct effect of one variable on another, indicated by the single‐headed arrows in Figure 2, is determined by the path coefficient *P_j_*, which is simply a standardized, partial regression coefficient. The squared path coefficient gives the fraction of the variance of the *Y* (dependent) variable that can be accounted for by the variance of the *X* (independent) variable and is a measure of the direct influence of *X* on *Y*. Indirect effects occur when two *X* variables are correlated (*r_ij_*), resulting in one *X* variable affecting the dependent variable through its relationship with another *X* variable (denoted by curved double‐headed arrows). Path analysis revolves around the path diagram shown in Figure 2. This analytical model is built around a specific set of causal relationships among traits that determine fitness (Scheiner, Mitchell, & Callahan, 2000). The residual, *U*, accounts for experimental error and variables not included in the model.

The formula for calculating the path analysis is as follows: Assuming there are *j* factors *x*_1_, *x*_2_, *x*_3_… *x_j_* and a single dependent variable *y*, *P_i_* is the coefficient of the direct path from *x_i_* to *y*, *r_ij_* is the correlation coefficient between any two factors, and r_ij_*P_j_ is the coefficient of the indirect path from factor *x_i_* to *y* via factor *x_j_*. The total correlation coefficient between *x_i_* and *y* is thus *r_iy_*, *i*, *j* = 1, 2, …, *p*.

(1) $$\left\{\begin{array}{c} P_{1}+r_{12}P_{2}+\cdots +r_{1j}P_{j}=r_{1y}\\ r_{21}P_{1}+P_{2}+\cdots +r_{2j}P_{j}=r_{2y}\\ \vdots \vdots \vdots \\ r_{j1}P_{1}+r_{j2}P_{2}+\cdots +P_{j}=r_{\mathrm{jy}} \end{array}\right.$$

To clarify which variable(s) play a decisive role or have a major restrictive effect on *y*, we calculated the decision coefficient. The decision coefficient from factor *x_i_* to *y* is $R_{\left(i\right)}^{2}$, and the formula is as follows (Wright, 1934; Yuan, Yu, Cai, Qin, & Li, 2001):

(2) $$R_{\left(i\right)}^{2}=P_{i}^{2}+2\sum_{j\neq i}P_{i}r_{\mathrm{ij}}P_{j}=R_{i}^{2}+\sum_{j\neq i}R_{\mathrm{ij}}^{2}$$

If the decision coefficient (*R* ^2^) is positive, the independent variable acts to promote the dependent variable; if *R* ^2^ is negative then the independent variable acts to impede the dependent variable.

There is a lack of consensus on which variables and environment factors are the most appropriate for use in analyses of seedling establishment. All of the variables we selected have been shown to have a relationships to seedling regeneration in previous studies (Doležal et al., 2009; Narukawa & Yamamoto, 2002; Takahashi, Mitsuishi, et al., 2003; Takahashi, Uemura, et al., 2003; Takanishi et al., 2002; Zheng et al., 2004). (a) Seed bank (SB). This is the seed source for seedling emergence; the seed bank was measured twice per year, in April (before seed germination) and in November (after seed rain). Many scholars have suggested that seed rain (seed production) should be used as the seed source in research on seedling establishment. However, we do not think that seed rain is appropriate for our study because, in the process of seed rain formation, there will be some natural seed dispersal (e.g., predispersal, postdispersal, animal predation, etc.), and these ecological processes may be significantly altered by the density of dwarf bamboo (Qian et al., 2016). Thus, using seed rain (seed production) would lead to greater errors in the seed germination data. Instead, we propose that it is more reliable to use seeds from the seed banks as the provenance for seedling establishment. The seeds in the seed bank are mainly dead, dormant, and germinating. Just like in sowing experiments, we can determine the number and species composition of seeds by investigating the demographics of the seed bank. (b) Leaf area index of the forest canopy (LAI). This refers to the total leaf area of plants per unit land area. The larger the LAI, the greater the degree of leaf overlap. In the energy flow of an ecosystem, light energy is mainly absorbed and transformed by plant leaves so the LAI (an important biological indicator of light absorption) is directly related to the efficiency of light capture. It is a comprehensive index of both the canopy structure and the utilization of light energy by vegetation. (c) Leaf area index of the dwarf bamboo canopy (LAID). (d) Soil temperature (ST). (e) Soil humidity (SH; soil water content). (f) Thickness of the litter (THL). (g) Thickness of the soil humus (THSH). After litter decomposition, humus is deposited on the soil surface. The thickness of soil humus represents its nutrient content. It is generally believed that the thicker the humus, the higher the nutrient content. (h) Dwarf bamboo density (relative density: 0%, 25%, 50%, and 100%). The environmental factors were all measured at the same time during each seedling survey.

In a preliminary analysis of the data, we attempted to construct multiple regression models using stepwise regression techniques (Bowers, Sonoda, & Mitchell, 1990) to predict seedling emergence and death from the independent variables (environment factors, seed bank, and bamboo density). We identified some significant models, but further analysis indicated that the independent variables in the various models were often highly correlated (Table 2). The regression coefficients therefore did not necessarily reflect the inherent effect of an independent variable on seedling emergence and death, but only a marginal effect, given whatever other correlated independent variable(s) were included in the models (Bowers et al., 1990). Thus, the environmental factors affecting seedling emergence and death could not be determined from regression analyses using stepwise model‐building techniques because of the confounding effect of multicollinearity, which resulted in an inability to validate the models and poor predictive ability of the models.

**Table 2:** Correlation coefficients for all variables

Items	LAI	LAID	THL	THSH	ST	SH	SB	ES	DS
BD	0.231*	0.992**	0.989**	0.953**	−0.897**	0.407**	−0.198*	−0.502**	−0.523**
LAI	1	0.241*	0.218*	0.186**	−0.222*	0.087	0.131	0.056	0.047
LAID		1	0.984**	0.953**	−0.886**	0.358**	−0.216*	−0.508**	−0.528**
THL			1	0.971**	−0.866**	0.362**	−0.165	−0.452**	−0.472**
THSH				1	−0.744**	0.284**	−0.139	−0.349**	−0.367**
ST					1	−0.579**	0.115	0.557**	0.582**
SH						1	0.452**	0.057	0.029
SB							1	0.806**	0.784**
ES								1	0.999**

Abbreviations: BD, bamboo density; DS, dead seedlings; ES, emerged seedlings; LAI, leaf area index of forest canopy; LAID, leaf area index of dwarf bamboo canopy; SB, seed bank (the number of active seeds in the seed bank); SH, soil humidity; ST, soil temperature; THL, thickness of litter; THSH, thickness of soil humus.

* *p* < .05.

** *p* < .01.

## 3 RESULTS

### 3.1 Effects of dwarf bamboo on the overall species composition and abundance of seeds and seedlings

At the community level, the seed density of the seed bank was not significantly different among the different bamboo density treatments (*F* = 1.074, *df* = 3, *p* = .366, Table 3 and Table S1). The average density of emerged seedling in plots without bamboo was 164.33 individuals per m^2^ per year, which was significantly higher than the average density in plots with bamboo (*F* = 8.391, *df* = 3, *p* < .01). However, there was no significant difference in the average number of surviving seedlings in the different plots (*F* = 1.383, *df* = 3, *p* = .316). Regardless of bamboo density, the average seedling emergence rate was generally low and was not significantly different among the different bamboo density treatments (*F* = 0.288, *df* = 3, *p* = .833). The average seedling death rate was significantly lower in the 100% density bamboo plots than in the 25% and 50% density bamboo plots (*F* = 3.321, *df* = 2, *p* = .044), but not significantly different from the bamboo‐free plots (*F* = 3.550, *df* = 1, *p* = .068). Moreover, although understory dwarf bamboo had a detrimental effect on seedling survival in some species, it was conducive to seedling survival in other species. Of note, high‐density dwarf bamboo tended to preferentially promote survival of evergreen seedlings, whereas low‐density dwarf bamboo tended to preferentially promote survival of deciduous seedlings (Table S1).

**Table 3:** Relationships between bamboo density and seedling emergence, seedling survival, and seed bank abundance

Items	0%	25%	50%	100%
Seed bank^a^	2,088.95 ± 1,006.52 a (37)	1,222.42 ± 394.31 a (40)	1,330.44 ± 409.82 a (33)	1,141.46 ± 394.68 a (35)
Emerged seedlings^a^	164.33 ± 24.42 a (33)	80.75 ± 11.08 b (33)	76.04 ± 12.17 b (33)	78.64 ± 3.55 b (28)
Surviving seedlings^a^	15.74 ± 2.77 a (11)	7.76 ± 1.41 a (15)	8.20 ± 1.24 a (14)	7.31 ± 0.14 a (13)
Emergence rate	0.186 ± 0.133 a	0.105 ± 0.060 a	0.090 ± 0.053 a	0.104 ± 0.052 a
Death rate	0.936 ± 0.021 ab	0.942 ± 0.024 b	0.940 ± 0.027 b	0.924 ± 0.019 a

Note

(1) For the seed bank, only active seeds were counted toward the total seed abundance. (2) The numbers in parentheses indicate the number of species. (3) Within each row, there was no significant difference (*p* < .05) between values marked by the same lowercase letter. (4) For the surviving seedlings, the data were subjected to cosine conversion because the variance was not homogeneous.

a The average density of seeds or seedlings (individuals per m^2^ per year, mean ± *SE*).

### 3.2 Dynamics of seedling emergence and survival in bamboo plots of differing density

**Figure 3:**
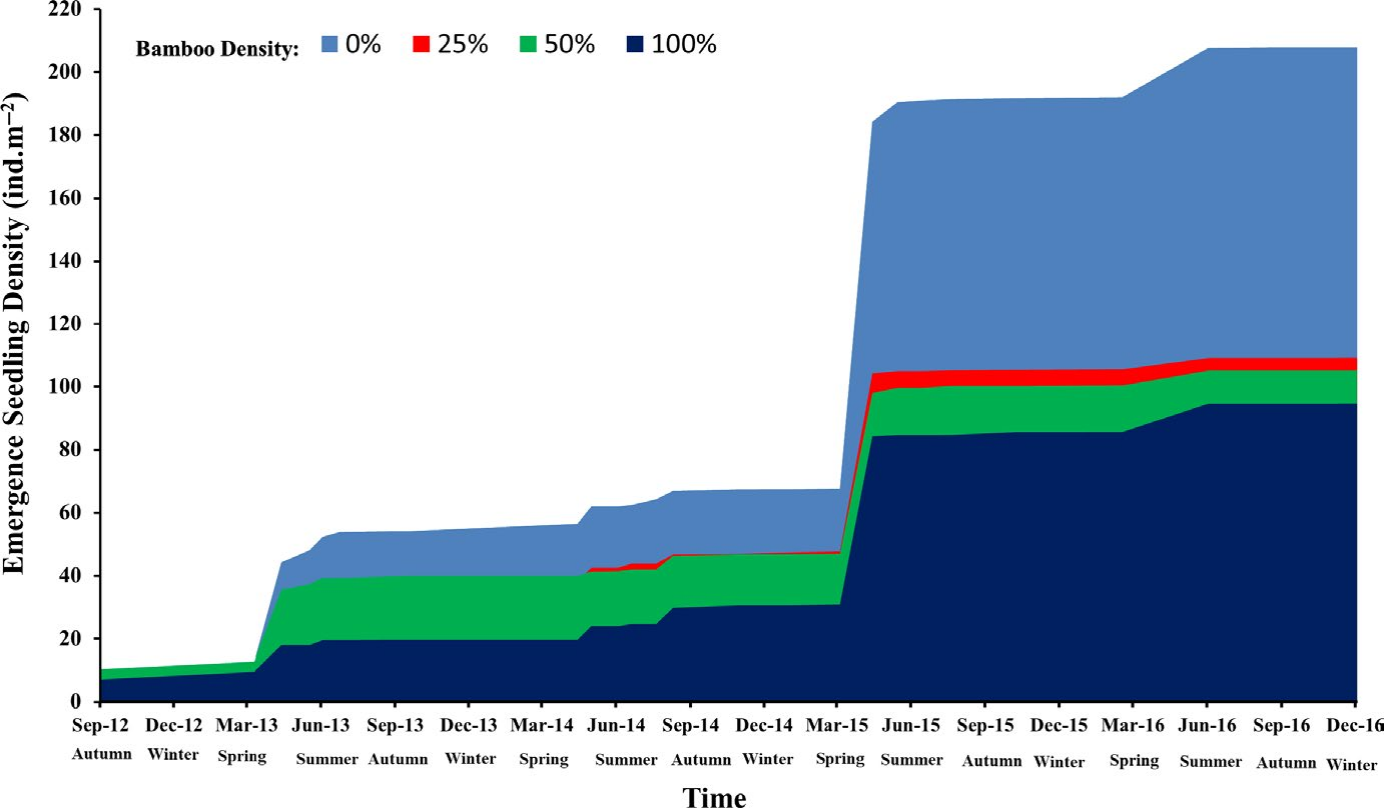
Accumulation curve for seedling emergence in dwarf bamboo plots of different density. Seedling emergence was recorded from 15 September 2012 to 17 December 2016, at approximately 15‐day intervals during the growing and drought seasons (March to August), and monthly during the other seasons

**Figure 4:**
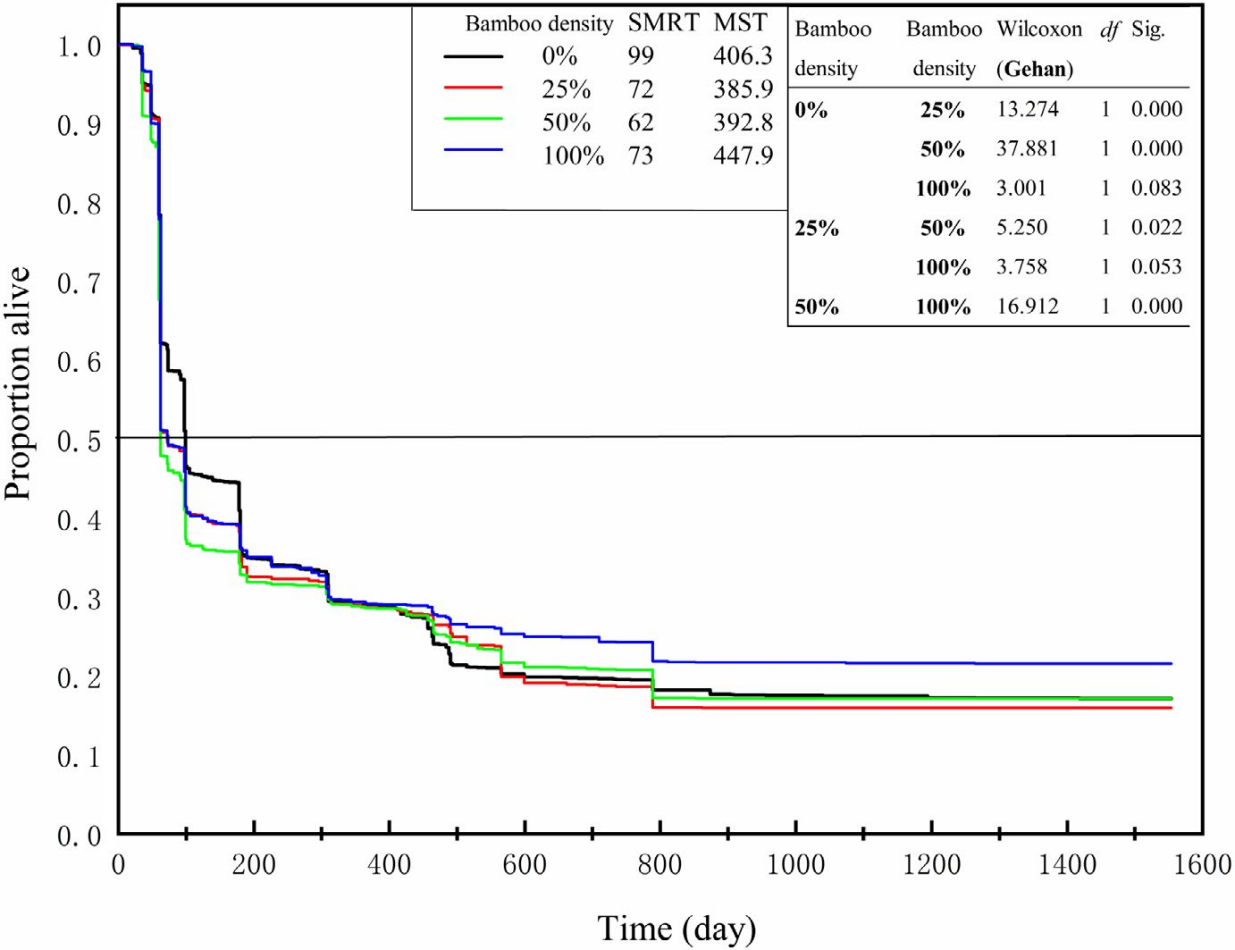
Seedling survival functions for different densities of dwarf bamboo. Survival analysis with life tables and the results of the pairwise Wilcoxon test (Gehan test) comparing the seedling survival functions across bamboo plots. MST, seedling mean survival time; SMRT, seedling median retention time (death speed)

During the five years of field observations, seedling emergence showed strong seasonal and annual fluctuations (Figure 3). Seedling emergence was greater in the spring, and 2015 had a greater density of emerging seedlings than the other years. More seedlings germinated in the plots without bamboo than the plots with bamboo. We conducted a survival analysis to explore the process of seedling death and found that the median retention times were 99, 72, 62, and 73 days, respectively, in plots with bamboo densities of 0%, 25%, 50%, and 100% (Figure 4). This demonstrates that, overall, the presence of dwarf bamboo accelerates the initial speed of seedling death, regardless of the density of the bamboo. However, the mean seedling survival times were 406.3, 385.9, 392.8, and 447.9 days, respectively, in plots with bamboo densities of 0%, 25%, 50%, and 100%. This demonstrates that dense (100%) dwarf bamboo is beneficial to the longer‐term survival of seedlings.

### 3.3 Relationship between dwarf bamboo density and microenvironmental factors

The average leaf area index of the forest canopy was not significantly different between the plots (*p* > .05). The average leaf area index of the dwarf bamboo canopy (and thus the light blocked by the bamboo canopy) differed significantly between plots with different bamboo densities (*F* = 2066.731, *df* = 3, *p* < .000) and increased with increasing bamboo density, reaching 12.14 in the densest plots (Table 4). The average thickness of litter and humus also increased with increasing bamboo density. Soil temperature (quantified at a depth of 5 cm) was significantly higher in plots without bamboo than in plots with bamboo (*F* = 46.257, *df* = 3, *p* < .001), decreasing with increasing bamboo density.. Correlation analyses showed that bamboo density was strongly correlated with LAID, THL, THSH, and SH (*p* < .01, Table 4).

**Table 4:** Mean values for all environmental factors in bamboo plots of different densities

Variable	Dwarf bamboo density			
	0%	25%	50%	100%
Leaf area index	3.35 ± 0.02 a	3.35 ± 0.02 a	3.34 ± 0.02 a	3.36 ± 0.02 a
Leaf area index of dwarf bamboo canopy	3.07 ± 0.05 a	5.19 ± 0.05 b	8.48 ± 0.19 c	12.14 ± 0.14 d
Thickness of litter (cm)	2.67 ± 0.01 a	3.03 ± 0.07 b	3.21 ± 0.04 c	4.05 ± 0.03 d
Thickness of soil humus (cm)	2.37 ± 0.06 a	2.45 ± 0.06 a	2.79 ± 0.05 b	4.05 ± 0.01 c
Soil temperature (°C)	24.78 ± 0.03 a	24.13 ± 0.03 b	23.72 ± 0.03 c	23.62 ± 0.02 c
Soil humidity (%)	44.85 ± 0.04 a	45.09 ± 0.08 a	45.15 ± 0.08 a	45.29 ± 0.08 a

Note

Within each row, different letters in the same row indicate statistically significant differences at *α* < 0.05 (one‐way ANOVA, with LSD multiple comparisons tests:* p* < .05).

### 3.4 Path analysis of relationships between environmental variables and seedling emergence and death

**Figure 5:**
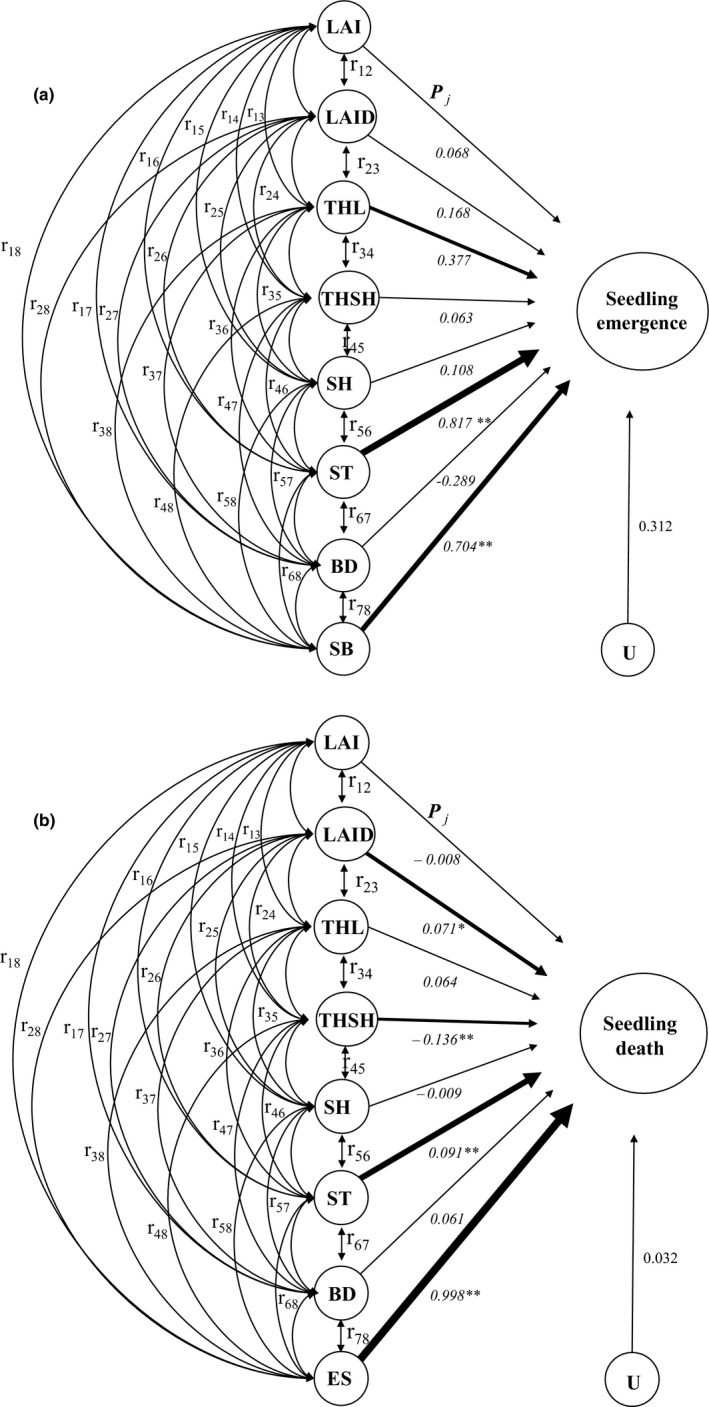
Path diagram illustrating the paths of influence of the independent variables (X) on the dependent variables (seedling emergence and seedling death). Environmental variables consisted of the leaf area index of forest canopy (LAI), the leaf area index of the dwarf bamboo canopy (LAID), the thickness of litter (THL), the thickness of soil humus (THSH), soil temperature (ST), soil humidity (SH), and bamboo density (BD: 0%, 25%, 50%, and 100%) The seed bank (SB) and the density of emerged seedlings (ES) were additional independent variables. *U* is the residual and *P_j_* = direct path coefficients. The strength of each path is indicated by the standardized partial regression coefficient and depicted by the thickness of the arrow. *r_ij_* = correlation coefficients

Instead, we used path analysis to quantify simultaneously the direct and indirect contributions of environmental factors to seedling emergence and death (Table 5, Figure 5). The variable with the greatest direct effect on seedling emergence was ST, followed by SB, THL, BD, LAID, SH, LAI, and THSH; only ST and SB had significant positive direct effects (*p* < .01). BD had a negative direct effect on seedling emergence but no significant (*P*_7 _= −0.289, *p* = .601, Figure 5a). However, LAID, THL, and THSH had significant negative indirect effects on seedling emergence through their effects on other variables (*p* < .05). SB and ST had the largest positive decision coefficient for seedling emergence (0.639 and 0.243), indicating that these were the main factors promoting seedling emergence; THL and LAID had the largest negative decision coefficients (−0.483 and −0.199), indicating that these were main factors limiting seedling emergence.

**Table 5:** Path analysis of the relationships between the emergence or death of seedlings and the various independent variables (environmental and biotic)

Factors	Direct effect	Indirect effect									Total correlation	Decision coefficients	Residual PU
		BD	LAI	LAID	THL	THSH	ST	SH	SB	Total			
Emerged seedlings													
BD	−0.289	…	0.016	0.167	0.373	0.06	−0.733	0.044	−0.139	−0.213	−0.502*	0.207	0.312
LAI	0.068	−0.067	…	0.04	0.082	0.012	−0.181	0.009	0.092	−0.012	0.056	0.003	
LAID	0.168	−0.287	0.016	…	0.371	0.06	−0.724	0.039	−0.152	−0.676***	−0.508*	−0.199	
THL	0.377	−0.286	0.015	0.165	…	0.061	−0.708	0.039	−0.116	−0.829***	−0.452*	−0.483	
THSH	0.063	−0.275	0.013	0.16	0.366	…	−0.608	0.031	−0.098	−0.412**	−0.349*	−0.048	
ST	0.817***	0.259	−0.015	−0.149	−0.326	−0.047	…	−0.063	0.081	−0.260	0.557*	0.243	
SH	0.108	−0.118	0.006	0.06	0.136	0.018	−0.473	…	0.318	−0.051	0.057	0.001	
SB	0.704***	0.057	0.009	−0.036	−0.062	−0.009	0.094	0.049	…	0.102	0.806*	0.639	

Factors	Direct effect	Indirect effect									Total correlation	Decision coefficients	Residual PU
		BD	LAI	LAID	THL	THSH	ST	SH	ES	Total			
Dead seedlings													
BD	0.061	…	−0.002	0.07	0.063	−0.13	−0.082	−0.004	−0.501	−0.584***	−0.523*	−0.068	0.032
LAI	−0.008	0.014	…	0.017	0.014	−0.025	−0.02	−0.001	0.056	0.055	0.047	−0.001	
LAID	0.071**	0.061	−0.002	…	0.063	−0.13	−0.081	−0.03	−0.507	−0.599***	−0.528*	−0.080	
THL	0.064	0.06	−0.002	0.07	…	−0.132	−0.079	−0.003	−0.451	−0.536***	−0.472*	−0.065	
THSH	−0.136***	0.058	−0.001	0.068	0.062	…	−0.068	−0.003	−0.348	−0.231**	−0.367*	0.081	
ST	0.091***	−0.055	0.02	−0.063	−0.055	0.101	…	0.005	0.556	0.491***	0.582*	0.098	
SH	−0.009	0.025	−0.01	0.025	0.023	−0.039	−0.053	…	0.057	0.038	0.029	0.001	
ES	0.998***	−0.031	−0.0004	−0.036	−0.029	0.047	0.051	0.001	…	0.001	0.999*	0.998	

Note

(1) Dwarf bamboo density (BD) was divided into four treatments with relative densities of 0%, 25%, 50%, and 100%; (2) Environmental data were measured at the same time that seedlings (emergence and death) were counted. (3) The thickness of soil humus (THSH: cm) and the thickness of litter (THL: cm) were measured with vernier calipers. (4) Seed bank (SB: ind. Y^−1^.m^−2^) data were collected once in 2012, then twice a year from 2013 to 2016, in April (before seed germination) and in November (after a large number of seeds have fallen to the surface).

Abbreviations: LAI, leaf area of the forest canopy; LAID, leaf area of the dwarf bamboo canopy; SH, soil humidity (%); ST, soil temperature (°C).

* The correlation is significant at the *p* < .01 level (*t* test).

** Significance of the path coefficient (direct or indirect effects) at the *p* < .05 level.

*** Significance at the *p* < .01 level (*t* test).

The density of emerged seedlings (ES) had the highest direct effect on seedling death (*P*_8_ = 0.998, Table 5, Figure 5b), followed by THSH, ST, LAID, THL, BD, SH, and LAI. ES, ST, and LAID had significant positive direct effects on seedling death (*p* < .01 for ES and ST, *p* < .05 for LAID), whereas THSH had a significant negative direct effect on seedling death (*p* < .01). LAID, BD, THL, and THSH had significant negative indirect effects on seedling death (−0.599, −0.584, −0.536, and −0.231), and ST had a significant positive indirect effect on seedling death (0.491). The decision coefficient for ES (0.998) was far higher than that for the other variables, demonstrating that ES was the key factor influencing seedling death, with the other factors merely ancillary.

## 4 DISCUSSION

### 4.1 Effects of dwarf bamboo on the understory microenvironment

The presence of a well‐developed dwarf bamboo understory modifies the abiotic and biotic environments of the forest floor in several ways. The density of dwarf bamboo branches and leaves significantly affects the intensity of light reaching the ground (Taylor et al., 2006). Konno (2001) showed that the intensity of incident light passing through a dense bamboo canopy and reaching the ground was less than 1%. The litter layer below dwarf bamboo is deeper than in bamboo‐free plots because of the large amount of litter fall from bamboo; also, dwarf bamboo has a negative effect on litter decomposition (Watanabe, Fukuzawa, & Shibata, 2017). The litter layer beneath dwarf bamboo may therefore act as a mechanical barrier. However, if there is no dwarf bamboo to trap and to hold litter, the soil humus is easily washed away by rainfall so the humus layer is thinner in bamboo‐free plots. A thinner insulating litter layer also contributes to elevated soil temperatures (Facelli & Pickett, 1991). In contrast to the research areas used in previous studies of the effects of a dwarf bamboo understory on light and litter structure, our research area was wet and perennially rainy, hence dwarf bamboo cover did not affect the soil water content. In terms of the biotic environment, dense dwarf bamboo provides habitat and shelter for predators of seeds and seedlings, which can lead to reduced seedling density (Wada, 1993).

### 4.2 Seedling emergence

Our analysis provides convincing evidence that dwarf bamboo reduces the density of emerged seedlings through its effects on the understory microenvironment. Soil temperature was an important direct factor for seedling emergence (Table 5) because a suitable soil temperature promotes the speed of water absorption, enzymatic processes, and seed respiration and accelerates the transformation of the endosperm into a soluble state (Bewley & Black, 1978). In natural communities, the fallen seeds mostly enter the litter layer or soil to form an overwintering seed bank and then germinate the following spring when the temperature rises and water is sufficient (Benvenuti, 1995; Kyereh et al., 2010). Light was an indirect factor for seedling emergence in our study. Gul and Weber (1998) reported that the seeds of some species require light to trigger germination after completing the transition from primary to secondary dormancy during the burial period. Our path analysis shows that light (LAID) did not directly contribute to seedling emergence in our study, but did indirectly influence seedling emergence through its effects on soil temperature. In our study, soil moisture was not an important factor for seedling emergence (decision coefficient: 0.001, path coefficient: 0.108). However, this does not mean that soil humidity is not important for seedling emergence (Takahashi, Mitsuishi, et al., 2003; Takahashi, Uemura, et al., 2003). Instead, our result is likely because the abundant rainfall in our study area provided sufficient and suitable water conditions for seed germination, so water was not a limiting factor for seedling germination in our study. The depth of the litter layer is a relatively easily neglected factor that affects seedling emergence (*r* = −.452, *p* < .01). As mentioned above, a thick litter layer not only reduces the possibility of seed germination by preventing seeds from reaching the soil surface, but it also hinders seed germination by altering the microclimate, nutrient cycle, and chemical allelopathy of the microenvironment (Faith, 1998; Jane & Carol, 1992). In forest understories with dwarf bamboo, the litter layer significantly limits successful seedling emergence (decision coefficient: −0.483). Our correlation analysis demonstrates that bamboo density is strongly related to light (LAID), soil humidity, and the thickness of litter and soil humus, and all of these factors have a significant influence on seedling emergence. Therefore, we suggest that understory dwarf bamboo not only directly affects the emergence and establishment of seedlings, but also indirectly changes the surface microenvironment in ways that affect seedling regeneration.

Our path analysis showed that various factors affect seedling emergence and that there are complex indirect relationships among these factors. According to the size of the decision coefficient, the key factor promoting seedling emergence at the community level was the seed bank (decision coefficient: 0.639). Tree seeds are an important biotic basis for forest regeneration (Harper, 1977), and the seed density determines the seedling germination potential. Soil temperature was an important factor promoting seedling emergence but thick litter layers hindered seedling emergence. In general, dwarf bamboo appears to have a notable effect on seedling emergence at sites where potential seedling emergence is high, as a results of high seed input or favorable environmental conditions. In parallel, whether seedlings became successfully established was largely dependent on the habitat conditions.

### 4.3 Seedling establishment

During our 5‐year investigation, most of the seedlings died within a short period of time and did not survive through the first growing season (Figure 4). The main reasons for this are likely summer wilting and herbivory of seedlings growing under the dwarf bamboo understory, whereas herbivory from rodents in seedlings in the bamboo‐free plots (Doležal et al., 2009). The results of our survival analysis showed that dwarf bamboo accelerated the speed of seedling death. It was likely because most seeds are stored in the litter layer. Plots with bamboo have thicker litter layers so the seedling roots cannot penetrate the litter layer rapidly to reach the soil beneath, thus exhausting their reserves and dying quickly (Edwin et al., 1996; Peterson & Facelli, 1992). Interestingly, the densest bamboo was beneficial to the mean survival time of seedlings (447 days). Seedlings that survived through the first growth season in the dense plots (with roots that managed to penetrate the soil) went on to survive for a comparatively long time because of the relatively stable environmental conditions in the dense bamboo plots, suited to the longer‐term survival and growth of seedlings (Doležal et al., 2009).

Many other studies have shown that the presence of a dwarf bamboo understory inhibits the density of surviving seedlings (Doležal et al., 2009; Itô & Hino, 2007; Narukawa & Yamamoto, 2002; Takahashi, Mitsuishi, et al., 2003; Takahashi, Uemura, et al., 2003; Tomimatsu et al., 2011), Consistent with this, we found a trend toward an increased density of surviving seedlings in bamboo‐free plots than in bamboo‐covered plots, but this trend was not significant. Also, most of the emerged seedlings died during the course of the experiment. We found significant effects of the dwarf bamboo on seedling death when analyzing across all seedlings in the community: the seedling death rate was lowest in the high‐density (100%) bamboo plots, followed by the bamboo‐free (0%) and medium‐density (50%) plots, and highest in the low‐density (25%) bamboo plots. This indicates that high‐density bamboo has a protective effect on seedling establishment, whereas low‐density and medium‐density bamboo have a negative effects. Moreover, evergreen species were lost more often in the bamboo‐ free and low‐density bamboo plots, whereas deciduous species were lost more often in the bamboo‐free and medium‐density bamboo plots. These differential effects on the death rate represent a mechanism by which the dwarf bamboo understory may act as a selective filter that influences future forest composition.

Bai et al. (2012) suggested that differences in seedling survival are caused by biotic and abiotic environmental factors. George and Bazzaz (1999) further proposed that, in systems where dominant understory species occupy different microsites, the activity of an understory filter must be interpreted in the context of this environmental heterogeneity. Therefore, in order to determine which factor(s) play the major role(s) in seedling establishment in forests with a bamboo understory, the variables to be studied must be selected according to the heterogeneity particular to dwarf bamboo forests. The results of our path analysis demonstrate that seedlings are more likely to be impacted by the biotic factors than by the abiotic factors, and the density of emerged seedlings was the key factor determining seedling death at the community level (decision coefficient: 0.998). We found a density‐dependent mortality effect: greater the density of emerged seedlings, the greater the seedling death. These changes show that there is a community compensatory trend in tree seedling survival, to some extent, in forests dominated by dwarf bamboo. This may contribute to the spatial dynamics of seedling recruitment (Bai et al., 2012). And the soil temperature and light (LAID) were the most important environmental factors for seedling survival in our study, consistent with the results reported by Campanello, Gatti, Ares, Montti, and Goldstein (2007).

## 5 CONCLUSION

In forest communities, successful establishment of seedlings is crucial for maintaining stable tree populations. Both the species composition and the quantity of seedlings can affect the dynamics of future forest communities. Seedling regeneration is strongly influenced by multiple biotic and abiotic factors. Our results present convincing evidence that understory dwarf bamboo obstructs tree seedling emergence, but contributes to the longer‐term survival of seedlings. In our study, the density of dwarf bamboo also affected which seedling species thrived: high‐density dwarf bamboo was beneficial to evergreen species but lower‐density of bamboo was conducive to the survival of deciduous species. Moreover, although bamboo reduced the median retention time of seedlings, dense bamboo increased the mean survival time of seedlings. These results show that understory dwarf bamboo has multiple selectivities for seedling emergence and establishment, which can alter the successional trajectory of forest communities.

Various factors affected seedling emergence, and there were also complex indirect relationships among these factors. Biological factors had a stronger effect on seedling germination and death than abiotic factors: at the community level, the most important factor for seedling emergence was the number of seeds, and the density of emerged seedlings was the key factor for seedling death. Among abiotic factors, increased soil temperature promoted seedling emergence but increased seedling death, and the thickness of the litter layer was the limiting factor for seedling emergence. LAID was the limiting factor for seedling death.

In the path analysis models, the path coefficients of the remaining factors were 0.312 (*P*_UE_) and 0.032 (*P*_UD_). This shows that the effect of other variables on seedling emergence—such as seed characteristics, seed dormancy, and the physical and chemical properties of the soil—was not taken into account in our field survey (Finch‐Savage & Leubner‐Metzger, 2006; Qian et al., 2016; Rachele, Davidjp, & Jamesw, 2010; Tripathi et al., 2005). This serves as a reminder that a more comprehensive set of variables should be considered in future studies.

## CONFLICT OF INTERESTS

We certify that we have participated sufficiently in the work to take public responsibility for the appropriateness of the experimental design and method, and for the collection, analysis, and interpretation of the data. All authors have reviewed the final version of the manuscript and approved it for publication. To the best of our knowledge and belief, this manuscript has not been published in whole or in part nor is it being considered for publication elsewhere.

## AUTHORS' CONTRIBUTION

Jianping Tao designed experiments, Miao Chen, Jiaqin Zeng and Chenqiang Dang carried out experiments, Haiyan Song analyzed experimental results, Feng Qian wrote the manuscript.

## Data Availability

We agree to deposit the data and analysis data to a public repository: http://osf.io/qe5tb.
