# Supplementary material for: Multivariate path analysis of the relationships between seedling regeneration and environmental factors beneath a dwarf bamboo understory
Source: Ecol Evol. 2019 Sep 3;9(18):10277–90. doi: 10.1002/ece3.5548 (PMC6787829; doi:10.1002/ece3.5548)
Supplement: Supplementary file 1 [file ECE3-9-10277-s001.docx]

**Table S1** Demographics of seeds and seedlings (individuals per m^2^ per year) in the different bamboo-density treatments (0%, 25%, 50%, 100%). Species names in bold font indicate evergreen species; species names in upright font indicate deciduous species. Seed data were derived from the number and species composition of active seeds in the seed bank.

|  |  | **Seed bank（active seeds）** | | | | **Emerged seedlings** | | | | **Surviving seedlings** | | | |  |
| --- | --- | --- | --- | --- | --- | --- | --- | --- | --- | --- | --- | --- | --- | --- |
| *Genus* | *Species* | 0% | 25% | 50% | 100% | 0% | 25% | 50% | 100% | 0% | 25% | 50% | 100% |  |
| *Cupressus Linn.* | ***Cupressus funebris* Endl.** | 4.93±5.62 | 0.08±0.12 |  | 0.21±0.30 |  |  |  |  |  |  |  |  |  |
| *Ilex Linn.* | ***Ilex chinensis* Sims** |  |  |  |  |  | 0.01±0.12 |  |  |  |  | 0.06±0.04 |  |  |
| *Pieris D. Don* | ***Pieris japonica* (Thunb.) D. Don ex G. Don** | 5.93±0.66 | 0.86±0.61 | 2.33±0.72 | 3.68±0.26 | 0.20±0.28 | 0.05±0.07 |  |  |  |  |  |  |  |
| *Rhododendron L.* | ***Rhododendron longipes* Rehd. et Wils var. *chienianum* (Fang) Chamb. ex Cullen et Chamb.** | 5.42±7.67 | 0.778±0.57 | 0.65±0.91 | 0.33±0.47 |  |  |  |  |  |  |  |  |  |
|  | ***Rhododendron coeloneurum* Diels** | 27.24±32.90 | 4.81±0.75 | 1.22±0.16 | 2.65±0.02 |  |  |  |  |  |  |  |  |  |
|  | ***Rhododendron calophytum* Franch. var. *jinfuense* Fang & W. K. Hu** | 6.11±3.66 | 0.13±0.18 |  | 0.51±0.59 |  |  |  |  |  |  |  |  |  |
|  | ***Rhododendron decorum* Franch. subsp. *parvistigmaticum* W. K. Hu** | 16.68±22.63 |  |  |  |  |  |  |  |  |  |  |  |  |
|  | ***Rhododendron maculiferum* Franch.** |  |  |  |  |  |  |  |  |  |  |  |  |  |
|  | *Rhododendron simsii* Planch. |  | 17.750±14.24 |  | 39.51±17.80 |  |  | 0.13±0.19 |  |  | 0.06±0.04 |  |  |  |
| *Vaccinium* | *Lyonia ovalifolia* (Wall.) Drude var. *elliptica* (Sieb.et Zucc.) Hand.-Mazz. | 0.94±0.49 | 1.92±2.24 |  | 0.65±0.55 | 0.79±1.12 | 3.44±4.73 | 2.07±2.78 | 0.95±0.95 |  | 0.06±0.04 | 0.06±0.04 |  |  |
| *Elaeocarpus* | *Elacocarpus japonicus* S. et Z. | 2.23±1.63 | 1.97±0.90 | 8.319±5.601 | 2.05±0.54 | 0.95±0.92 | 0.83±0.78 | 0.36±0.37 | 0.28±0.26 |  |  |  |  |  |
| *Betula Linn.* | *Betula luminifera* H. Winkl. |  | 0.78±0.57 |  | 0.78±0.57 |  |  |  |  | 0.03±0.04 | 0.03±0.04 |  |  |  |
| *Carpinus Linn.* | *Carpinus viminea* Lindley | 1442.17±935.67 | 821.52±402.80 | 919.18±414.85 | 795.71±445.84 | 63.00±46.49 | 34.33±14.06 | 32.29±18.51 | 36.54±28.39 | 9.58±1.14 | 3.38±0.49 | 3.55±0.25 | 2.94±1.84 |  |
| *Cyclobalanopsis Oerst.* | ***Cyclobalanopsis glauca* (Thunberg) Oersted** | 1.56±2.21 | 1.02±1.44 |  |  |  | 0.13±0.09 |  |  |  |  |  | 0.06±0.04 |  |
| *Lithocarpus* | ***Lithocarpus glaber* (Thunb.) Nakai** |  | 3.33±4.71 |  | 2.60±3.54 | 0.50±0.71 | 0.29±0.41 | 0.71±1.00 | 0.21±0.30 |  |  |  |  |  |
| *Quercus L.* | *Quercus serrata* Murray | 0.08±0.12 | 0.88±1.24 |  | 0.06±0.09 | 0.13±0.19 |  |  |  |  |  |  |  |  |
| *Vitex L.* | *Vitex negundo* L. |  |  | 0.44±0.63 |  |  |  |  |  |  | 0.03±0.04 |  |  |  |
| *Ligustrum Linn.* | *Ligustrum sinense* Lour. |  |  |  |  | 0.13±0.19 | 0.63±0.88 | 0.17±0.24 | 0.19±0.15 |  |  |  | 0.12±0.08 |  |
| *Pistacia L.* | *Pistacia weinmanniifolia* J. Poisson ex Franchet |  |  |  |  | 0.42±0.59 | 0.92±1.30 | 0.86±1.08 | 0.48±0.34 |  |  |  |  |  |
| *Acer Linn.* | *Acer davidii* Franch. | 7.64±1.12 | 16.46±7.91 | 9.88±4.80 | 21.60±10.95 | 3.83±5.42 | 0.83±1.18 | 0.33±0.47 | 0.08±0.12 | 0.15±0.03 |  |  | 0.06±0.08 |  |
| *Photinia Lindl.* | ***Photinia prunifolia* (Hook. et Arn.) Lindl.** | 2.02±1.53 | 2.94±2.15 |  | 0.78±0.57 | 5.12±6.38 | 2.47±3.21 | 3.17±4.48 | 1.13±0.89 | 0.20±0.08 | 0.23±0.08 | 0.46±0.20 | 0.14±0.04 |  |
| *Pyracantha* | ***Pyracantha fortuneana* (Maxim.) Li** |  |  |  |  | 2.38±2.38 | 0.81±0.43 | 0.77±0.21 | 0.76±0.12 |  |  |  |  |  |
| *Rubus L.* | *Rubus lambertianus* Ser. |  |  |  |  | 1.25±1.77 | 0.28±0.26 | 0.50±0.25 | 0.32±0.32 |  | 0.06±0.04 |  |  |  |
| *Sorbus L.* | *Sorbus folgneri* (Schneid.) Rehd. | 1.37±1.04 | 2.42±0.80 | 1.37±1.04 | 3.05±1.21 | 0.07±0.09 |  | 0.08±0.12 |  | 0.06±0.04 | 0.03±0.04 | 0.03±0.04 |  |  |
| *Viburnum Linn.* | ***Viburnum chinshanense* Graebn.** |  |  |  |  | 0.31±0.21 | 0.20±0.16 | 0.07±0.09 | 1.04±1.47 |  |  |  |  |  |
|  | *Viburnum dilatatum* Thunb. |  |  |  |  | 0.63±0.88 |  | 0.29±0.41 | 0.25±0.35 | 0.27±0.07 | 0.23±0.04 | 0.09±0.01 | 0.38±0.19 |  |
|  | *Viburnum erosum* Thunb. | 1.22±0.16 | 0.33±0.47 | 2.59±1.03 | 1.22±0.16 | 0.80±0.75 | 0.00±0.59 | 0.91±0.79 | 1.33±1.25 |  |  |  |  |  |
|  | *Viburnum setigerum* Hance |  |  |  |  | 14.79±20.92 | 0.08±0.12 |  |  |  |  |  |  |  |
| *Cephalotaxus* Sieb. et Zucc. ex Endl. | ***Cephalotaxus fortunei* Hooker** |  |  |  |  | 1.29±1.83 | 0.17±0.24 | 0.63±0.88 | 0.33±0.47 |  |  |  |  |  |
| *Ficus Linn.* | *Ficus heteromorpha* Hemsl. |  |  |  |  |  | 0.13±0.19 | 0.13±0.18 |  |  |  |  |  |  |
| *Camellia L.* | ***Camellia japonica* L.** |  |  |  |  |  |  |  |  |  |  |  |  |  |
|  | ***Camellia rosthorniana* Handel-Mazz.** |  |  |  |  | 1.42±2.00 | 1.04±1.47 | 1.46±2.06 | 0.17±0.24 |  | 0.09±0.01 |  | 0.03±0.04 |  |
|  | ***Camellia sinensis* (L.) O. Ktze.** |  |  |  |  | 1.21±1.03 | 0.20±0.16 |  | 0.20±0.28 |  |  |  |  |  |
| *Eurya Thunb.* | ***Eurya japonica* Thunb.** | 27.97±13.94 | 59.83±31.82 | 56.03±23.54 | 23.27±13.58 |  |  |  |  |  |  | 0.12±0.08 |  |  |
|  | ***Eurya loquaiana* Dunn** |  | 0.78±0.57 | 4.63±0.62 | 4.76±3.39 | 3.40±4.67 | 4.08±5.78 | 7.41±10.06 | 3.20±4.10 |  |  |  |  |  |
|  | ***Eurya nitida* Korthals** | 0.33±0.47 | 0.86±0.61 | 3.38±1.55 | 3.11±0.56 |  |  |  |  |  |  |  |  |  |
| *Symplocos Jacq.* | ***Symplocos lancifolia* Sieb. et Zucc.** | 1.49±1.08 | 0.80±0.76 | 1.22±0.16 |  |  |  |  |  |  |  |  |  |  |
|  | ***Symplocos setchuensis* Brand** | 14.53±6.12 | 1.22±0.16 | 0.08±0.12 | 1.98±1.42 | 3.08±4.36 | 0.46±0.65 | 0.233±0.21 | 0.38±0.53 |  |  |  |  |  |
|  | ***Symplocos sumuntia* Buch.-Ham. ex D. Don** | 2.73±2.22 | 7.96±1.38 | 0.65±0.91 | 12.30±6.39 | 4.38±6.19 | 1.42±2.00 | 0.71±1.00 | 0.46±0.65 | 0.14±0.08 | 0.26±0.01 | 0.55±0.08 | 0.55±0.08 |  |
| *Dendrobenthamia Hutch.* | *Cornus capitata* Wallich | 30.11±17.65 | 35.96±40.48 | 23.03±14.03 | 39.49±23.24 | 1.13±0.58 | 0.96±0.06 | 5.83±4.99 | 3.84±3.04 |  |  |  |  |  |
| *Euscaphis Sieb.et Zucc.* | *Euscaphis japonica* (Thunb.) Dippel | 338.11±354.64 | 97.09±34.32 | 117.94±54.34 | 68.97±28.22 | 1.67±2.36 | 0.71±1.00 | 0.92±1.29 | 0.04±0.06 | 4.99±2.612 | 3.09±1.755 | 2.86±1.59 | 2.77±1.71 |  |
| *Acanthopanax Miq.* | *Acanthopanax gracilistylus* W. W. Smith |  |  |  |  | 42.90±26.98 | 17.48±17.21 | 10.62±10.17 | 14.38±17.71 |  |  |  |  |  |
| *Aralia Linn.* | *Aralia elata* (Miq.) Seem. |  |  |  | 0.33±0.47 |  |  | 0.13±0.18 |  |  |  |  |  |  |
| *Nothopanax Miq.* | *Metapanax davidii* (Franchet) J. Wen & Frodin | 62.41±61.53 | 34.57±15.61 | 85.81±54.00 | 23.03±4.23 |  |  | 0.07±0.09 |  |  |  | 0.06±0.04 | 0.06±0.04 |  |
| *Cinnamomum Trew* | ***Cinnamomum wilsonii* Gamble** |  |  |  |  | 2.83±3.57 | 1.54±2.18 | 1.28±1.66 | 1.99±2.41 |  |  | 0.06±0.04 | 0.03±0.04 |  |
| *LitseaLam.* | ***Litsea elongata* (Wall. ex Nees) Benth. et Hook. f. var. *subverticillata* (Yang) Yang et P.H.Huang** | 5.85±2.78 | 1.37±1.04 | 2.39±1.33 |  | 1.26±1.51 | 0.71±1.00 | 0.55±0.52 | 2.83±3.30 |  |  |  |  |  |
|  | *Litsea pungens* Hemsl. |  |  |  |  |  | 0.07±0.09 |  |  |  |  |  |  |  |
| *Neolitsea Merr.* | ***Neolitsea pulchella* (Meissn.) Merr.** | 10.24±9.36 | 4.59±4.40 | 5.52±3.61 | 11.44±5.49 | 0.11±0.08 |  | 0.07±0.09 |  | 0.06±0.04 | 0.06±0.04 | 0.06±0.04 |  |  |
|  | ***Neolitsea aurata* (Hay.) Koidz. var. *glauca* Yang** | 1.96±2.77 | 2.72±0.72 | 1.50±0.86 | 0.98±0.06 | 1.05±1.21 | 1.34±1.62 | 0.84±0.92 | 2.98±4.08 |  | 0.14±0.20 | 0.20±0.04 | 0.09±0.01 |  |
| *Ardisia* | ***Ardisia crispa* (Thunb.) A. DC.** |  |  |  |  | 1.73±2.31 | 3.67±4.49 | 1.85±1.37 | 3.56±4.47 |  |  | 0.06±0.08 |  |  |
|  | ***Ardisia crenata* Sims** | 1.22±0.16 | 0.78±0.57 | 1.22±0.16 |  | 0.75±1.06 | 0.33±0.47 | 0.21±0.29 | 0.13±0.18 | 0.07±0.10 | 0.03±0.04 |  | 0.09±0.01 |  |
|  | *unidentified 1* | 2.22±3.14 | 2.00±0.54 | 1.22±0.16 |  | 0.84±0.92 | 0.27±0.26 | 0.44±0.49 | 0.61±0.01 |  |  |  |  |  |
|  | *unidentified 2* |  | 1.22±0.16 | 2.34±0.28 | 1.22±0.16 |  |  |  |  |  |  |  |  |  |
|  | *unidentified 3* | 30.27±18.58 | 72.03±37.67 | 50.99±30.74 | 57.56±31.03 |  |  |  |  |  |  |  |  |  |
|  | *unidentified 4* | 5.45±6.67 |  | 11.44±11.01 |  |  |  |  |  |  |  |  |  |  |
|  | *unidentified 5* | 7.36±0.52 | 0.25±0.35 | 1.89±2.01 | 7.75±5.80 |  |  |  |  |  |  |  |  |  |
|  | *unidentified 6* | 3.47±0.58 | 6.42±1.10 | 2.27±0.68 | 2.94±2.15 |  |  |  |  |  |  |  |  |  |
|  | *unidentified 7* | 3.51±2.65 | 5.94±1.02 | 3.93±1.16 | 3.80±2.75 |  |  |  |  |  |  |  |  |  |
|  | *unidentified 8* | 7.57±3.77 | 0.89±1.26 | 3.00±1.18 | 1.57±1.12 |  |  |  |  |  |  |  |  |  |
|  | *unidentified 9* | 3.42±4.84 | 3.94±1.03 | 1.35±0.02 | 0.51±0.59 |  |  |  |  |  |  |  |  |  |
|  | *unidentified 10* | 1.57±1.12 | 3.23±1.37 | 1.33±0.27 |  |  |  |  |  |  |  |  |  |  |
|  | *unidentified 11* | 1.59±1.13 | 1.31±1.10 | 1.33±0.27 | 1.31±1.04 |  |  |  |  |  |  |  |  |  |
